# Gender differences in recreational and transport cycling: a cross-sectional mixed-methods comparison of cycling patterns, motivators, and constraints

**DOI:** 10.1186/1479-5868-9-106

**Published:** 2012-09-08

**Authors:** Kristiann C Heesch, Shannon Sahlqvist, Jan Garrard

**Affiliations:** 1 Queensland University of Technology, Institute of Health & Biomedical Innovation and the School of Public Health and Social Work, Brisbane, QLD, Australia; 2 Medical Research Council Epidemiology Unit and UKCRC Centre for Diet and Activity Research (CEDAR), Institute of Public Health, Forvie Site, Cambridge, UK; 3 School of Health and Social Development, Deakin University, Burwood, VIC, Australia

**Keywords:** Bicycling, Gender, Exercise, Physical activity, Active transport, Health promotion

## Abstract

**Background:**

Gender differences in cycling are well-documented. However, most analyses of gender differences make broad comparisons, with few studies modeling male and female cycling patterns separately for recreational and transport cycling. This modeling is important, in order to improve our efforts to promote cycling to women and men in countries like Australia with low rates of transport cycling. The main aim of this study was to examine gender differences in cycling patterns and in motivators and constraints to cycling, separately for recreational and transport cycling.

**Methods:**

Adult members of a Queensland, Australia, community bicycling organization completed an online survey about their cycling patterns; cycling purposes; and personal, social and perceived environmental motivators and constraints (47% response rate). Closed and open-end questions were completed. Using the quantitative data, multivariable linear, logistic and ordinal regression models were used to examine associations between gender and cycling patterns, motivators and constraints. The qualitative data were thematically analyzed to expand upon the quantitative findings.

**Results:**

In this sample of 1862 bicyclists, men were more likely than women to cycle for recreation and for transport, and they cycled for longer. Most transport cycling was for commuting, with men more likely than women to commute by bicycle. Men were more likely to cycle on-road, and women off-road. However, most men and women did not prefer to cycle on-road without designed bicycle lanes, and qualitative data indicated a strong preference by men and women for bicycle-only off-road paths. Both genders reported personal factors (health and enjoyment related) as motivators for cycling, although women were more likely to agree that other personal, social and environmental factors were also motivating. The main constraints for both genders and both cycling purposes were perceived environmental factors related to traffic conditions, motorist aggression and safety. Women, however, reported more constraints, and were more likely to report as constraints other environmental factors and personal factors.

**Conclusion:**

Differences found in men’s and women’s cycling patterns, motivators and constraints should be considered in efforts to promote cycling, particularly in efforts to increase cycling for transport.

## Background

Cycling offers health benefits, including improved cardio-respiratory fitness and decreased risk of all-cause and cardiovascular mortality [1]. Commuter cycling is negatively associated with overweight and obesity [2] and may help employees meet physical activity recommendations of moderate- to vigorous-intensity physical activity for 30 minutes per day, 5 days per week [3]. Active travel is also good for the environment as it can reduce traffic congestion, air and noise pollution, carbon emissions and fossil fuel consumption [4].

In Australia, only about 1% of daily trips are by bicycle, similar to the percentages seen in the US and the UK, but low compared with the 26% of daily trips reported for the Netherlands and 9-18% for some other European countries [5]. However, cycling for recreation is the fourth most commonly-reported physical activity among Australian adults [6]. The most recent data indicate that 17% of Australian adults cycled in the previous month but that most cyclists (62%) cycled only for recreation [7]. Given that recreational cyclists already possess the motivation, equipment and skill, it has been argued, that in countries like Australia with low rates of transport cycling, recreational cyclists might comprise a useful target group for promoting cycling for transport [8].

Addressing gender differences in cycling and the reasons for these differences will be important for increasing transport cycling in countries such as the USA, UK and Australia. In Australia, not only do more men than women cycle in general [7], but even among cyclists, more men cycle for transport. In the state of Queensland, we have found that only 24% of transport cyclists are women [9]. In Sydney, only 17% of bicycle commuting trips are by women and in Melbourne, only 25% are [10]. Although these percentages are comparable to those in the US, where 24% of commuting trips are by women, they are much lower than in countries with established cycling cultures, such as the Netherlands and Germany, where women cycle at similar rates to men [5,11].

Gender differences in transport cycling in Australia and other car-dependent countries reflect in part the different transportation patterns, needs, and purposes of men and women [12,13]. For example, issues of safety, comfort and accessibility to destinations appear to be more important to women’s overall travel behavior than to men’s [14]. This may partly explain the low transport cycling rates for women, as studies have found that women are more likely than men to report safety concerns as constraining their transport cycling [15]. Gender differences may also be explained by the nature of a typical transport cycling journey in Australia. The average cycle commute trip length is high, 10 km in Queensland [9] and 11-15 km in Melbourne [15], generally higher than seen in Europe [16]. Such trips may appeal to the most motivated, fit and sporty recreational cyclists, as the commute to work becomes an opportunity to improve fitness; however, the long distances may discourage other cyclists and women disproportionately so. International data indicate that women are more likely than men to trip chain as part of their commute, given their responsibilities for transporting children and other household members and to do the household shopping [12]. These tasks require different cycling equipment and cycling style to those which are common in countries such as Australia [10].

Although gender differences are noted in travel patterns in general and in transport cycling specifically, studies have tended to make broad comparisons, and few studies have focused on modeling male and female cycling patterns separately [13,17]. Given the low prevalence of transport cycling in countries like Australia, this modeling is difficult to achieve in studies of the general population as so few people report cycling. Studies which explicitly sample cyclists can provide valuable data on gender differences in cycling behavior. The primary aims of this study were to examine, in a population of current cyclists, gender differences in cycling patterns and in motivators and barriers to cycling, separately for recreational and transport cycling. A secondary aim was to explore possible overlaps in cycling patterns between recreational and transport cycling to better understand gender differences in cycling for different purposes. Most of the data collected to address our aims were quantitative; however, some qualitative data were gathered to expand the knowledge obtained from the quantitative findings.

## Methods

### Sampling and study protocol

Adult cyclists residing in Queensland were administered an online survey to assess their attitudes, behaviors and cycling experiences. The sampling frame was the adult membership (aged ≥18 years) of Bicycle Queensland (BQ), a state-wide community organization that promotes recreational and transport cycling, organizes community bike rides for all levels of cycling ability and advocates for better cycling facilities and improved safety (see bq.edu.au). A small proportion of members are competitive cyclists. As found for Australian cyclists more broadly [7], most members cycle only for recreation, with less than half cycling for transport [9].

BQ sent email invitations, with a link to the survey, to the ‘primary members’ of member households, to encourage all adult BQ members of the household to participate. One week later, BQ sent reminder emails. To further encourage participation, respondents could enter into prize draws to win bicycle accessories and receive the study findings. This study was conducted with the approval of The University of Queensland Human Research Ethics Committee.

As reported elsewhere [18], 2085 of 4469 invited households responded (47% response rate). These households included 2356 individual respondents. Of these respondents, those who did not complete the survey (n = 189), indicated that they were not members of BQ (n = 245) or reported a residence outside Queensland (n = 62) were excluded from analysis, leaving data from 1862 respondents available for inclusion in this analysis.

### Measures

Most questions were adapted from those used previously [15], although new demographic information questions were added to better characterize the sample and additional barriers were added to reflect the climate, topography and policies in Queensland.

#### Socio-demographic characteristics

Respondents completed standard demographic questions (sex, age, educational level, employment status, home postcode, body mass index) and details about their home environment, including the number of cars available for use, and the household composition. Home postcodes were used to determine socio-economic indexes for areas (SEIFA). This measure uses 2006 Census variables to assess the relative socio-economic advantage or disadvantage of Australian geographic areas [19], and for this study, respondents’ residential SEIFA. Areas are divided into deciles with higher deciles representing greater advantage. Using postal codes, respondents were also classified as living in a major city; inner regional area; or outer regional, rural or very rural area. Body mass index (BMI; kg/m^2^) was calculated from self-reported height without shoes and weight without clothes or shoes.

#### Cycling patterns

Respondents reported the length of time they had been cycling as an adult (weeks, months, years), their cycling frequency (5*–7 days/week* to *never in the last year*), and the purposes of their cycling (*recreation [just for fun or exercise]*, *competition*, and/or *transport [as a means of getting to and from places]*).

#### Transport cycling behavior

Respondents reported whether they rode for transport in the previous week. Those who responded *yes* reported the number of bicycle trips taken for transport in the previous week (*counting each single trip to a place as one trip and each return trip from a place as another trip)*, the total time spent cycling for transport in the previous week, and the destinations of these trips (*work, university/vocational school/school, shops, recreational venues, friends/relatives*). Respondents also described the bicycle routes they used in the last week and their route preferences given current traffic conditions and patterns. For these items, they selected one or more of three options: *off-road or shared pedestrian/bike paths*; *designated on-road bike lanes, such as the bicycle awareness lanes painted green*; and *on the road (no bicycle lanes).*

#### Recreational cycling behavior

Respondents reported whether they cycled for recreation in the previous week. Those who responded *yes* reported the number of recreational bicycle trips taken in the previous week and the total time spend cycling for recreation that week. They were instructed not to include any cycling reported already as transport cycling. Last, they reported the bicycle routes they used in the last week for recreational cycling and their route preferences, using the same response options included in the items asking about transport cycling routes used and preferred.

#### Motivating and constraining factors

In keeping with social-ecological models of behavioral determinants, respondents who had cycled for any purpose in the previous year were asked about personal, social and perceived environmental factors that were hypothesized to motivate or constrain cycling behavior, as identified in previous research [15]. Respondents rated the importance of 18 factors in motivating them to cycle, using a 4-point scale (*very important* to *not at all important*). These were dichotomized as important (*important* and *very important* = 1) or not important (*not at all* important and *slightly important* = 0). Respondents were also asked whether 20 factors made it difficult for them to cycle more. Responses were on a 4-point scale (*major constraint* to *not a constraint*). These were dichotomized as a constraint (*moderate* and *major constraints* = 1) or not a constraint (*minor constraint* or *not a constraint* = 0). Last, respondents reported in an open-ended response format any other constraints or difficulties that deterred them from cycling in their local area.

#### Physical activity

The Active Australia physical activity questions were used to assess physical activity (PA) levels. Respondents reported time spent in the last week (in ≥ 10-minute sessions) walking (*for recreation or exercise or to get to and from place to place*), and in moderate- and vigorous-intensity leisure-time physical activities, and they were asked to include their cycling in their responses. Using standard procedures [20], a total score was calculated as the sum of the minutes spent in each PA multiplied by an assigned metabolic equivalent value (MET): walking = 3.0 METs; moderate-intensity PA = 4.0 METs; vigorous-intensity PA = 7.5 METs, to account for differences in intensity among the activities. A summary score of ≥600 MET minutes per week is equivalent to 150 minutes per week of moderate-intensity PA, the cut-off for meeting Australian PA guidelines [21]. Thus, those reporting ≥600 MET minutes per week were considered to be meeting PA guidelines.

### Analysis

All quantitative analyses were conducted with STATA/SE 10.1 (StataCorp, College Station, Texas). Missing data were imputed using the Hotdeck procedure that uses all other available data to impute a value for categorical variables. The survey (svy) command was used to account for clustering of respondents within households (StataCorp, 2007). Descriptive statistics were generated for all quantitative study variables. Logistic and linear regression modeling was used to examine whether gender was associated with the transport and recreational cycling behavior variables, after adjusting for other demographic characteristics and for cycling patterns. For examining associations between gender and non-normal variables (times spend in each type of cycling and in total PA) the same modeling was performed except ordered logistic regression was used with the outcome variable categorized into quintiles. Moreover, given apparent duplication of transport and recreational cycling trips reported by some transport cyclists, recreational cycling modeling was limited to the subgroup of respondents who reported no transport cycling in the previous week. Significance was set at p < 0.05.

Two authors (KCH, SS) analyzed the open-ended survey responses. The qualitative data collected on usage of, and preferences for, cycling paths were used to place participants into the respective usage and preference categories already defined in the survey (e.g., any description of cycling away from roads was coded into the existing *off-road or shared pedestrian/bike paths* category) and to better describe these categories. KCH and SS each independently coded these data into the quantitative categories, and then discussed discrepancies between their coding before reaching consensus. The data collected on cycling constraints were used to expand our understanding of the barriers to cycling beyond the categories included in the questionnaire. For the first step, KCH and SS independently reviewed the qualitative constraint data to identify major themes. Next, they used these themes to independently code the constraint data and to look for any gender differences. Discrepancies between coders were discussed in team meetings and consensus was used to determine the final themes. As the final step in the analyses of all the qualitative data, KH summarized the findings in consultation with SS.

## Results

### Sample characteristics

Women comprised 29% of the sample. Compared with men, women tended to be significantly younger, more educated, less likely to be living with children, living in a household with one car, working part-time and of a normal weight (Table 1). The main purpose of cycling for both men and women was for recreation. Few men and women reported to cycle for transport only; instead, those who cycled for transport also tended to cycle for recreation. Most men and women were meeting PA guidelines.

**Table 1:** Characteristics of the total sample and men and women separately (n = 1862), Queensland, Australia

**Characteristics**	**Sample**	**% of total sample**^**a**^	**% of men**^**a**^	**% of women**^**a**^
	**n**	**%**	**%**	**%**
Age (years)				p = 0.0001
18-34	209	15.8	15.7	15.9
35-44	482	22.0	21.1	23.4
45-54	635	30.8	28.5	34.2
55-64	406	24.5	25.4	23.0
65+	130	6.9	9.3	3.5
Education				p = 0.021
No tertiary degree	266	14.4	14.0	14.9
Trade/apprenticeship or certificate/diploma	361	18.4	20.1	15.9
Undergraduate degree	628	34.1	35.2	32.5
Postgraduate university degree	607	33.1	30.7	36.7
Household				p <0.0001
Live alone	212	12.5	9.6	16.7
Live with adults and no children	971	55.2	53.9	57.1
Live with adults and children	679	32.3	36.4	26.3
No. of cars in household				p = 0.003
0	34	2.1	1.6	2.7
1	677	38.0	34.8	42.7
2	869	45.4	47.7	41.9
3+	282	14.6	15.9	12.6
Employment				p <0.0001
Full-time paid work	1,350	68.6	76.9	56.5
Part-time paid work	262	16.7	8.9	28.0
Retired or not in paid work	250	14.7	14.2	15.4
SEIFA				p = 0.18
Decile 10 (most advantaged)	516	27.9	27.6	28.3
Decile 9	563	29.5	31.5	26.8
Decile 8	332	18.0	18.2	17.8
Decile 7	157	8.8	7.9	10.1
Deciles 1-6 (most disadvantaged)	294	15.7	14.8	17.0
Residential location				p = 0.10
Urban	1,574	84.4	85.9	82.1
Inner suburban	186	10.1	8.9	11.9
Outer suburban or more remote	102	5.5	5.2	6.0
BMI				p <0.0001
Normal (BMI <25)	1,022	58.8	49.9	71.8
Overweight (BMI 25- < 30)	665	32.5	39.2	22.9
Obese (BMI ≥ 30)	175	8.7	11.0	5.3
Years of cycling as an adult				p <0.0001
10+ years	794	40.2	47.0	30.2
5 - < 10	428	23.9	21.0	28.2
2 - < 5	441	24.5	22.2	27.9
0 - < 2	199	11.4	9.7	13.8
Cycling frequency				p <0.0001
5-7 days/week	447	22.9	27.5	16.3
3-4 days/week	720	38.3	40.2	35.5
1-2 days/week	513	27.6	26.4	29.4
At least once/month	101	6.2	3.4	10.2
At least once in previous 3 months	45	2.7	1.7	4.1
At least once in the last year	36	2.3	0.8	4.5
Cycle purpose last week				p <0.0001
Recreation and transport	535	27.9	31.5	22.6
Recreation only	783	42.0	41.5	42.8
Transport only	292	15.6	16.5	14.4
Did not cycle last week	252	14.4	10.5	20.2
Total physical activity (mins/week)				p = 0.87
Not meeting guidelines	231	12.5	12.4	12.7
Meeting guidelines	1631	87.5	87.6	87.3

^a^ Percentages account for clustering of respondents within households. p-values refer to differences between men and women in proportions within categories of a variable, using Pearson’s chi-square.

### Transport cycling patterns

More men than women reported that they cycled for transport in the previous week (p < 0.0001; Table 2). This finding reflected the finding that more men than women cycled to their work or place of study (p < 0.0001; Table 2). Among the 827 cyclists who reported transport cycling in the previous week, time spent in transport cycling was more for the 625 men (proportional OR with males as referent = 0.68 [95%CI 0.49, 0.95]), with the median minutes for men (240 mins; inter-quartile range [IQR] 150, 360]) higher than for the 202 women (180 mins [IQR 105, 300]). However, there was no gender difference in the number of transport cycling trips taken in the previous week among these cyclists (b = -0.24; 95% CI = -0.53, 0.05; p = 0.10): after adjusting for demographic and cycling pattern variables, the mean number of cycling trips for men was 3.55 trips (95% CI 3.41, 3.69) and for women, 3.03 trips (95% CI 2.80, 3.25). There were also no gender differences in MET minutes of total PA for the previous week (proportional OR with males as referent = 0.86 [95% CI 0.62, 1.19]), with median MET minutes for men of 2460 (IQR 1320, 3960) and for women, 2085 (IQR 1215, 3510). The proportion of total PA from transport cycling also did not differ between genders (proportional OR with males as referent = 0.74 [95%CI 0.53, 1.04]), with a median proportion of PA from transport cycling for men of 57.1% (IQR 32.1% , 98.6%) and for women, 40.0% (IQR 20.0% , 80.0%).

**Table 2:** **Gender differences in transport and recreation cycling patterns in the previous week: results of multivariable analysis**^**a**^

	**% of men**	**% of women**	**Gender differences**^**b**^	
			**OR**	**95% CI**
Transport cycling in sample (n = 1862)	46.7	34.7	**0.58**	**0.45-0.76**
Destination				
Work or study	37.2	25.0	**0.55**	**0.41-0.72**
Shops	10.5	8.7	0.80	0.55-1.18
Recreation facilities	9.2	9.7	1.16	0.81-1.67
Friends	2.7	3.8	1.39	0.82-2.35
Transport cycling in the last week (n=827)				
Cycle routes used				
Off-road	**77.9**	**84.5**	**1.88**	**1.15-3.06**
On-road designated cycle lane	71.9	75.7	1.34	0.88-2.05
On-road	**91.7**	**87.6**	**0.58**	**0.34-0.99**
Cycle routes preferred				
Off-road	65.0	70.0	1.37	0.93-2.00
On-road designated cycle lane	74.3	75.1	0.96	0.64-1.42
On-road	**12.6**	**6.2**	**0.49**	**0.27-0.89**
Recreational cycling in sample (n = 1862)	**74.7**	**65.6**	**0.69**	**0.53-0.88**
Recreational cycling in the last week (n=1318)				
Cycle routes used				
Off-road	**56.9**	**66.7**	**1.66**	**1.24-2.23**
On-road designated cycle lane	58.1	62.7	1.17	0.86-1.56
On-road	**87.0**	**83.3**	**0.65**	**0.44-0.95**
Cycle routes preferred				
Off-road	**52.1**	**64.6**	**1.79**	**1.34-2.38**
On-road designated cycle lane	77.2	80.4	1.07	0.76-1.49
On-road	**32.5**	**16.5**	**0.37**	**0.26-0.52**

^a^All statistics account for clustering within household and adjust for age, education, employment status, household, number of cars in household, SEIFA, residential location, BMI, and years cycled as an adult,

^b^ Male is the referent category, Bold: p < 0.05.

Most transport cyclists used a combination of cycle routes. Based on the qualitative data, we included within the ‘off-road’ category bush paths (e.g., through parks) and footpaths. Qualitative responses also indicated that some cyclists qualified their responses on usage or preference for ‘on-road’ (e.g., cycling ‘on road shoulders’ or ‘on quiet streets only’). Women were more likely to use off-road paths (p = 0.011) whereas men were more likely to cycle on-road (p = 0.045). However, few men or women preferred to cycle on the road, with women less likely than men to prefer cycling on the road (p = 0.020). Interestingly, more men and women were cycling off-road than would prefer to do so. This may be explained in part by qualitative data indicating that respondents perceived that most off-road paths were not direct routes to destinations. One woman reported, “The most direct route is along major motorways that do not have any cycle path option, so consequently I have to ride kilometers out of the way.” It may also reflect the observation by respondents that off-road travel typically required sharing congested paths with pedestrians and animals. Not surprising then, our qualitative findings indicated a preference by many transport cyclists for dedicated cycle-only paths separated from both motorists and pedestrians.

### Recreational cycling patterns

More men than women cycled for recreation in the previous week (p = 0.003; Table 2). Among the 783 respondents who cycled for recreation but not for transport in the previous week, the time spent in recreational cycling was higher for the 553 men (proportional OR with males as referent = 0.64 [95%CI 0.47, 0.88]), with men spending a median of 279 mins (IQR 180, 420) and the 230 women, a median of 240 mins (IQR 180, 360). The men also took more recreational cycling trips than did the women (b = -.44 [95% CI = -0.68, -0.20]; p < 0.001]): the adjusted mean number of recreational cycling trips for men was 2.87 trips per week (95%CI 2.74, 3.01), and for women, 2.48 trips (95%CI 2.28, 2.64). As shown for transport cycling, for recreational cycling there were no gender differences in MET minutes of total PA (proportional OR with males as referent = 0.78 [95%CI 0.57, 1.08]), with a median MET minutes for men of 2760 [IQR 1725, 4140] and for women of 2880 [IQR 1710, 4230]). There was also no gender difference in the proportion of total PA from recreational cycling (proportional OR with males as referent = 0.73 [95%CI 0.52, 1.01]), with a median proportion of minutes from recreational cycling for men of 65.2% (IQR 40.0%, 87.5%) and for women of 52.7% (IQR 30.8%, 85.7%).

As found for transport cyclists, most recreational cyclists used a combination of paths. Based on the qualitative data, the off-road category included bush paths (e.g., called dirt, bush, forest, park, fire, mountain bike tracks), rail trails, the beach, and footpaths. Female recreational cyclists were more likely to use off-road paths (p = 0.001) while their male counterparts were more likely to cycle on-road (p = 0.025) (Table 2). Moreover, women were less likely than men to prefer cycling on-road (p < 0.001), but more likely to prefer cycling off-road (p < 0.001). More recreational than transport cyclists preferred cycling on-road although cycling on-road was the least preferred option among both types of cyclists.

As found for transport cyclists, the qualitative data indicated that many recreational cyclists preferred designated bicycle paths away from both motor vehicle and pedestrian traffic. Those who cycled for recreation on bush paths, rail trails, or the beach said these were the preferred paths as well. Some who preferred on-road cycling quantified their response (e.g., ‘on road in slower traffic’, ‘on road with wide shoulders,’ ‘quiet sealed country roads’, ‘early morning quiet roads’).

### Bicycling motivators

Top motivators for cycling for most men and women were personal factors related to health (improving or maintaining fitness, relaxing and reducing stress, building physical activity into a busy lifestyle) and enjoyment (fun and enjoyment, being a challenge, getting fresh air) with women significantly more likely than men to agree that fun and enjoyment, building physical activity into a busy lifestyle and getting fresh air were important motivators (Table 3). Women were more likely than men to agree that other motivators were important, including other personal factors (confidence in own cycling abilities), social factors (something active I can do with other people, seeing other people cycle, participating in a cycle event or program, encouragement from others) and perceived environmental factors related to transport (convenient or cheap form of transport, concerns about the environment).

**Table 3:** **Motivators for men and women to cycle, of total sample, transport-only cyclists, and recreation-only cyclists**^**a**^

**Motivators**	**Respondents who cycled within the last year (n = 1849)**^**b**^				**Respondents who only cycled for transport in the last week (n = 292)**^**b**^				**Respondents who only cycled for recreation in the last week (n = 783)**^**b**^			
	**Men**	**Women**	**Gender differences**		**Men**	**Women**	**Gender differences**		**Men**	**Women**	**Gender differences**	
	**%**	**%**	**OR**	**95%CI**	**%**	**%**	**OR**	**95%CI**	**%**	**%**	**OR**	**95%CI**
Improving / maintaining fitness	98.6	97.6	0.73	0.37-1.45	98.1	98.9	2.99	0.32-10.39	100	100	**^c^	** ^c^
Fun and enjoyment	88.6	91.2	**1.54**	**1.05-2.25**	70.6	79.9	1.87	0.83-4.19	95.0	95.0	1.12	0.47-2.68
Relaxation / stress reduction	87.4	83.6	0.90	0.65-1.23	78.1	79.2	1.72	0.78-3.83	92.2	88.3	0.71	0.40-1.27
Building physical activity into my busy lifestyle	85.4	90.0	**1.86**	**1.32-2.63**	89.5	91.7	0.99	0.35-2.80	86.0	91.0	**2.06**	**1.20-3.54**
To get outside in the fresh air	78.8	88.6	**2.26**	**1.64-3.12**	66.1	80.7	**2.86**	**1.29-6.34**	86.7	92.7	1.82	1.00-3.03
It is a challenge	70.0	71.1	1.10	0.86-1.42	43.3	50.8	1.33	0.71-2.50	78.7	82.9	1.50	0.93-2.42
It is a low impact activity	68.4	64.1	0.90	0.71-1.15	60.8	50.3	0.66	0.36-1.20	73.1	65.3	0.72	0.48-1.08
Time out to myself	65.5	67.6	1.22	0.96-1.57	49.0	59.5	1.80	0.91-3.57	72.0	69.0	0.93	0.62-1.39
Other health reasons	59.4	62.0	**1.28**	**1.01-1.62**	54.2	63.6	1.56	0.82-2.95	63.3	63.7	1.14	0.77-1.67
It is something active I can do with other people	57.8	68.9	**1.53**	**1.20-1.96**	21.4	26.6	1.14	0.57-2.29	70.7	82.9	**1.71**	**1.09-2.69**
It is a convenient form of transport^d^	57.6	64.2	**1.32**	**1.03-1.70**	90.5	93.7	1.29	0.38-4.33				
Concerns about the environment^d^	53.5	71.7	**2.02**	**1.58-2.59**	70.0	84.4	1.98	0.93-4.20				
Confidence in my cycling ability	51.2	62.6	**1.64**	**1.29-2.09**	45.8	45.7	1.03	0.54-1.98	55.7	66.9	**1.64**	**1.11-2.43**
It is a cheap form of transport^d^	46.5	63.8	**1.88**	**1.47-2.40**	82.9	94.6	**3.16**	**1.30-7.66**				
Seeing other people cycling	38.8	50.5	**1.48**	**1.17-1.87**	30.2	41.6	1.66	0.85-3.23	44.7	54.5	1.22	0.85-1.76
Participating in a cycling event or program like Ride to Work Day	38.5	47.7	**1.47**	**1.16-1.86**	22.4	25.6	1.15	0.58-2.27	40.7	52.2	**1.63**	**1.12-2.36**
Encouragement from family, friends or work colleagues	22.8	36.7	**1.73**	**1.34-2.23**	19.4	28.2	1.27	0.64-2.51	23.3	36.5	**1.66**	**1.13-2.45**
Encouragement from supervisors or employer^d^	10.0	18.0	**1.73**	**1.25-2.40**	10.2	18.9	2.54	1.10-5.84				

^a^ All statistics account for clustering within household and adjust for age, education, employment status, household, number of cars in household, SEIFA, residential location, BMI, and years cycled as an adult, ^b^ Male is the referent category, ^c^ Not computed due to lack of variability between genders in responses, ^d^ Statistics not computed for recreational cycling as these were hypothesized to pertain only to transport cycling, Bold: p < 0.05.

Top motivators for most male and female transport-only cyclists also included it being a convenient and cheap form of transport and having concerns about the environment, with women more likely than men to agree that cycling being a cheap form of transport and concerns about the environment were important motivators. For male and female recreation-only cyclists, the top motivators included the social aspect (something active I can do with other people), with the women were more likely to agree that this was a motivator.

### Bicycling constraints

Top constraints for at least half of the men and women were perceived environmental factors, namely traffic and aggression from motorists, with women significantly more likely than men to report these constraints (Table 4). Of transport-only and recreation-only cyclists, female recreation-only cyclists was the group with the most respondents reporting these constraints, and male transport-only cyclists was the group with the fewest respondents reporting them. Women were also more likely to report as constraints other perceived environmental factors related to traffic and transport issues (inhaling car fumes when cycling, inability to put a bicycle on public transport, living too far from destinations), weather and climate conditions (decreased in daylight hours during winter months, rain or story weather, windy weather, hot or humid weather, presence of hills), and individual factors (lack of fitness or confidence in abilities) (Table 4). Female transport-only cyclists was the group with the most respondents reporting the traffic and transport constraints, whereas female recreation-only cyclists was the group with the most respondents reporting weather and climate factors to be constraining, except for the presence of hills, which equally constrained female transportation-only and recreation-only.

**Table 4:** **Constraints on men’s and women’s cycling, of total sample, transport-only cyclists, and recreation-only cyclists**^**a**^

**Constraints**	**Respondents who cycled within the last year (n = 1849)**					**Respondents who only cycled for transport in the last week (n = 292)**			**Respondents who only cycled for recreation in the last week (n = 783)**			
	**Men**	**Women**	**Gender differences**^**b**^		**Men**	**Women**	**Gender differences**^**b**^		**Men**	**Women**	**Gender differences**^**b**^	
	**%**	**%**	**OR**	**95%CI**	**%**	**%**	**OR**	**95%CI**	**%**	**%**	**OR**	**95%CI**
Concerns about cycling in traffic	53.2	67.6	**1.6**	**1.27-2.05**	52.6	61.0	1.01	0.53-1.94	59.3	72.3	**1.53**	**1.04-2.52**
Aggression from motorists	52.6	86.8	**1.54**	**1.22-1.95**	44.9	60.8	1.62	0.86-3.04	52.2	67.0	**1.82**	**1.25-2.66**
Rainy or stormy weather	49.5	58.7	**1.28**	**1.02-1.62**	43.4	47.2	0.95	0.49-1.82	54.6	59.5	1.11	0.76-1.61
Lack of time	40.8	41.9	1.15	0.90-1.47	20.5	26.9	**2.50**	**1.26-4.96**	52.9	45.9	0.78	0.52-1.18
Lack of safe places to park or store my bicycle at places I would want to ride my bicycle to	37.7	40.0	1.03	0.81-1.30	29.3	38.9	1.50	0.78-2.87	40.8	47.3	1.12	0.77-1.63
Inhaling car fumes when cycling on the road	34.4	47.9	**1.63**	**1.29-2.07**	34.8	45.9	1.49	0.77-2.87	30.7	41.4	**1.66**	**1.13-2.43**
Lack of shower and changing facilities at places I would want to ride my bicycle to	28.3	33.0	1.18	0.92-1.52	23.3	28.1	0.98	0.51-1.90	32.9	41.4	1.44	0.97-2.15
Inability to put my bicycle on public transportation (buses, trains)	27.9	39.1	**1.54**	**1.20-1.96**	27.0	40.5	**2.49**	**1.06-5.81**	24.3	32.6	1.39	0.94-2.06
Decrease in daylight hours during winter months	25.7	38.9	**1.83**	**1.43-2.34**	10.1	22.9	**3.08**	**1.37-6.94**	35.2	42.1	1.22	0.84-1.77
Windy weather	19.7	37.3	**2.26**	**1.73-2.96**	11.5	22.1	2.09	0.92-4.74	21.7	41.3	**2.48**	**1.67-3.67**
Hot or humid weather	17.0	31.3	**1.91**	**1.46-2.51**	11.8	23.8	1.62	0.72-3.60	18.0	33.0	**1.97**	**1.28-3.04**
Living too far away from places I would want to ride my bike to	16.3	24.2	**1.70**	**1.28-2.26**	17.8	19.7	2.30	0.76-6.99	12.9	18.3	1.56	0.99-2.46
Cold weather	14.6	18.9	1.21	0.90-1.64	5.1	5.2	1.04	0.32-3.42	16.6	18.6	0.96	0.62-1.48
Illness, injury or health problems	13.4	16.5	1.23	0.90-1.68	7.4	13.6	2.26	0.94-5.41	10.9	14.1	1.40	0.86-2.30
The presence of hills	8.5	25.7	**3.98**	**2.88-5.49**	8.8	22.6	**3.97**	**1.73-9.11**	8.2	23.0	3.60	2.11-6.14
Lack of knowledge about local cycling routes	8.2	17.0	2.01	1.41-2.87	3.3	11.4	2.42	0.95-6.19	9.2	15.2	**1.73**	**1.02-2.94**
Cost of cycling (bicycles, accessories, clothing, rides)	6.1	4.1	**0.54**	**0.32-0.90**	5.3	3.8	0.75	0.20-2.77	5.7	3.3	0.45	0.20-1.03
Lack of fitness	4.5	12.1	**3.21**	**2.12-4.85**	3.9	8.2	**3.10**	**1.16-8.29**	3.8	9.0	**2.51**	**1.28-4.93**
Lack of confidence in bicycle maintenance, such as repairing a puncture	2.2	20.2	**10.2**	**6.43-16.18**	1.9	14.6	**11.39**	**3.27-39.58**	1.5	16.7	**11.59**	**5.51-24.40**
Lack of confidence in my cycling ability or skills	2.2	14.3	**5.97**	**3.85-9.25**	0.5	7.3	**10.71**	**1.18-97.44**	2.6	13.5	**5.23**	**2.60-10.50**

^a^All statistics account for clustering within household and adjust for age, education, employment status, household, number of cars in household, SEIFA, residential location, BMI, and years cycled as an adult, ^b^ Male is the referent category, Bold: p < 0.05.

The qualitative data indicated that inadequate infrastructure was a major barrier for both men and women. Most importantly, respondents perceived that the infrastructure was unsafe for cycling. These data expand upon the quantitative findings that concern about cycling in traffic was the primary barrier for both men and women and for both recreational and transport cyclists, and the data support our findings about men’s and women’s cycle route preferences. One major infrastructural concern was the poor conditions of existing road and cycle paths. As one woman explained, “They just mark off a crappy, potholed gravel strewn section of the usual road and whack a picture of a bike on it and call it a bike lane.” Another woman explained that the city had “not really taken into account the way cycle traffic flows” in road design. As a consequence of the perceived inadequate cycling infrastructure, respondents reported that they encountered rough surfaces, uneven and little maintained road shoulders, and that “rural roads [that] are very third world with being narrow, pitted and cracked [with] loose verges.” The other major safety concern was that the infrastructure made interactions with motorists on-road and pedestrians and animals off-road unavoidable. Respondents reported concern with the narrowing of bicycle access over bridges and roundabouts, the lack of safe crossings for cyclists across heavy traffic, and, most mentioned, “disconnects between pathways.” Respondents in rural areas in particular described the “near lack” of on-road cycle paths or bicycle paths. Such concerns with the roads help explain the earlier finding that more men and women were cycling on the road than would prefer to do so. Sharing paths with pedestrians was also cited as a barrier as these paths were reported to be congested at certain times of day with pedestrians often not aware of other path users. This finding may in part explain why the quantitative data collected about cycling route preferences indicated that more cyclists were cycling off-road than would prefer to do so. Moreover, many respondents reported danger from animals. A few described attacks from dogs or issues with other animals (snakes, wild pigs, dingos) crossing paths or roads, making travel by bicycle unsafe. The main animal culprit however were magpies and other nesting birds that would attack cyclists venturing near their nests during early summer.

## Discussion

In this sample of Queensland, Australia cyclists, both recreational and transport cycling was predominately undertaken by highly educated, full-time employed, middle-aged men and for recreation. This finding supports other Australian data showing that most cycling is by middle-aged men and for recreation [7] and is consistent with data from Melbourne and from other car-dependent countries showing women cycle less for transport [11,12,15,17,22-25] and for recreation [26]. In contrast, in countries with high rates of cycling, cycling rates are similar between men and women [5,11].

The gender difference in transport cycling was due to men’s greater likelihood of commuter cycling. In contrast, in the Netherlands, a high cycling country, women are just as likely to cycle to work as men [3]. Our findings may reflect women’s lower willingness to cycle the relatively long commuting distances in Australia, with constraints such as climate and weather factors, poor fitness levels, and lack of confidence in bicycle maintenance and in their own cycling skills compounding travel distance for women. Men’s and women’s low and similar rates of cycling to non-work destinations have also been found in Melbourne [15] and likely reflect the bicycle infrastructure in Australia, which supports longer work commutes from suburbs to urban centers [27]. The low rates may also reflect the key motivation for men and women to cycle, fitness, which may encourage some cyclists to take advantage of opportunities for long bicycle trips to work, but discourage their taking shorter trips for other purposes. In contrast, a study in Minnesota (US) found that women are more likely than men to cycle for non-commute trips [17]. In Tokyo, where men and women report high rates of weekly cycling, women are only half as likely as men to bicycle to work but are more likely to cycle for non-commute trips [28]. Thus, the focus in Australian capital cities on providing commuter cycling routes into city centers, while neglecting cycling infrastructure in suburban areas, may be constraining transport cycling in general, and women’s participation in transport cycling in particular.

Our study showed that, on average, both male and female transport and recreational cyclists are exceeding physical activity guidelines [21], with the average time spent cycling for either purpose exceeding 200 minutes per week. Likewise, findings from a national Australian survey indicate that cyclists accumulate over 200 minutes of physical activity per week [7]. Thus, Australian cyclists are an active subgroup compared with the general Australian population, 57% of whom are meeting physical activity guidelines [29]. In contrast, when transport cycling is socially inclusive, as in many high-cycling countries, population subgroups that often have low levels of physical activity (e.g., women) are more likely to achieve adequate levels of physical activity [30-34].

As found in the state of Victoria [15] and in cities in Canada [12] and the US [17,35,36], men and women in this study preferred cycling routes separated from motorists. Our study adds that on-road routes were even less preferred for transport cycling than recreational cycling by both men and women, possibly because recreational cyclists can choose the day and time of their cycling and thus can ride when roads are quieter, whereas transport cyclists may have less choice, particularly for commuting to work.

This study also adds to what we know about motivators and constraints to cycling in Australia. As in other Australian states [15,37,38] and elsewhere [3,22,24], top motivators for cycling were related to health, fitness and enjoyment. These were important motivators for men and women and for transport and recreational cyclists, although women tended to perceive other factors to be motivating as well. Motivators for transport cycling also included perceptions about the cost and convenience of bicycle travel, and about the environment, which have been documented previously [15,22,24,37-39]. Our study adds that these factors are more motivating to women than to men. In contrast, in high-cycling countries such as the Netherlands, cycling is more commonly seen as an appealing, convenient and safe alternative to car travel in urban areas by men and women, with the health and exercise benefits more incidental than deliberative [10].

Whereas in countries with good bicycling infrastructure most barriers tend to be personal [3,40], infrastructure and other environmental barriers were clearly important constraints in our sample. The key constraints were related to traffic conditions, motorist aggression, and safety, consistent with barriers reported for other Australian cities [15,37,41,42] and in studies from the UK and US [22,24,43-46], as is the finding that safety is more of a concern for women than men. Findings that personal, social, and policy factors constrained cycling also support previous literature [15,22,24,25,47,48]. Our study further showed that women perceived more constraints, and some differences were noted between female transport-only and recreation-only cyclists that reflect cycling purpose, most notably, that traffic and transport factors were important constraints to more female transport-only cyclists and weather and climate factors were important constraints to more female recreation-only cyclists.

Strengths and limitations of the study should be considered. Strengths included the mixed method design, unusual for survey studies; the relatively large sample, which allowed for a detailed examination of gender differences in two types of cycling; and the inclusion of a large number of potential correlates, for statistical control of socio-demographic variables that have not been previously examined in studies of gender differences in cycling [12,15] but that are known correlates of physical activity [49]. The major limitation was the sampling from a cycling community group, which may have resulted in a sample of respondents who were more experienced and motivated cyclists than other samples of cyclists and thus may have exhibited different cycling behaviors, motivators and barriers from those of other cyclists. Comparisons with Australian data on cyclists [6] indicate that our findings are biased towards middle-aged adults and slightly biased toward men. The sample characteristics do reflect that in Australia, cycling is predominantly undertaken by middle-aged men [7], and the sample included a good cross-section of different types of riders. Our sample also tended to be of relatively high socio-economic status, which supports travel data from elsewhere [50-52] that suggest a socio-economic gradient in transport cycling in Australian and other car-dependent English-speaking countries. It should also be noted that our response rate of 47% is low but excellent for an online survey [53] and is comparable or better than response rates found for some recent large population-based studies in Australia [7,54,55]. Other limitations include the reliance on cross-sectional self-report data that only captured cycling patterns, behaviors and perceptions at one point in time and were subject to recall bias.

## Conclusion

Our findings provide evidence of a substantial overlap between recreational and transport cycling in Australia. Namely, almost all transport-only cyclists, both male and female, reported that fitness improvement or maintenance was their main motivation for transport cycling; these cyclists were primarily cycling only to a destination (work) far enough away from home to allow for fitness training; and both transport and recreational cyclists were highly physically active, with participation in either type of cycling making a substantial contribution to physical activity levels. We conclude that promoting transport cycling, particularly commuting cycling, to recreational cyclists, may increase cycling for transport, but most likely among men and the most athletic. With literature from the transport field indicating women choose their transport mode based on safety and accessibility [14], adoption of transport cycling by women will require conversion in Australian society to a *transport* cycling culture, one in which there is a strong commitment to prioritizing transport cycling over car travel for short daily trips; providing bicycle infrastructure and end of trip facilities to support short, safe and direct trips; and promoting everyday cycling in city and suburban neighborhoods. The findings from this study support prior work [8] that suggests that a strategy of creating system-wide networks of designated bicycle paths will assist in achieving higher levels of more socially-inclusive transport cycling. Our findings also suggest differences in men’s and women’s cycling patterns, motivators and constraints that should be considered in efforts to promote cycling. In summary, the establishment of cycling as a convenient, safe and enjoyable form of transport for a wide range of trip purposes in multiple settings is likely to increase the bicycle mode share of transport, and, in particular, encourage more women to go along for the ride.

## Competing interests

The authors declare that they have no competing interests. No external financial support received.

## Authors’ contributions

KCH participated in the study conceptualization and design, reviewed the literature, analyzed the data, participated in the interpretation of the findings, and drafted and revised the manuscript. SS participated in the study conceptualization and design, the analysis of the qualitative data, the interpretation of the findings, and revisions of the manuscript. JG participated in the study conceptualization and design, in the interpretation of the findings and in revising the manuscript. All authors have read and approved the final manuscript.
